# Metabolic Modulation and Potential Biomarkers of the Prognosis Identification for Severe Aortic Stenosis after TAVR by a Metabolomics Study

**DOI:** 10.1155/2020/3946913

**Published:** 2020-10-28

**Authors:** Yanbiao Liao, Chang Liu, Tianyuan Xiong, Mingyue Zhao, Wen Zheng, Yuan Feng, Yijian Li, Yuanweixiang Ou, Zhengang Zhao, Yong Peng, Jiafu Wei, Qiao Li, Wei Meng, Xiaojing Liu, Mao Chen

**Affiliations:** ^1^Department of Cardiology, West China Hospital, Sichuan University, 37 Guoxue Street, Chengdu 610041, China; ^2^Laboratory of Cardiovascular Diseases, Regenerative Medicine Research Center, West China Hospital, Sichuan University, 37 Guoxue Street, Chengdu 610041, China; ^3^Laboratory of Mitochondrial Biology, West China-Washington Mitochondria and Metabolism Center, West China Hospital, Sichuan University, No. 88, Keyuan South Road, High-Tech Zone, Chengdu 610093, China; ^4^Department of Cardiovascular Surgery, West China Hospital, Sichuan University, 37 Guoxue Street, Chengdu 610041, China

## Abstract

**Objectives:**

To investigate the metabolic profile in patients with aortic stenosis (AS) after transcatheter aortic valve replacement (TAVR) and explore the potential biomarkers to predict prognosis after TAVR based on metabolomics.

**Methods and Results:**

Fifty-nine consecutive AS patients were prospectively recruited. Blood samples from the ascending aorta, coronary sinus, and peripheral vein at before and after TAVR were collected, respectively. Liquid chromatography-mass spectrometry and gas chromatography-mass spectrometry were performed to analyze the metabolic profile before and after TAVR. Influential metabolites were identified by integrating the univariate test, multivariate analysis, and weighted gene coexpression network analysis (WGCNA) algorithm. PLS-DA analysis revealed a significant extremely early (within 30 minutes after TAVR) alterations of metabolites in the ascending aorta, coronary sinus, and peripheral vein. The early (within 7 days after TAVR) changed metabolites in the peripheral vein were involved in purine metabolism, primary bile acid biosynthesis, glycerolipid metabolism, amino sugar and nucleotide sugar metabolism, one carbon pool by folate and alanine, and the aspartate and glutamate metabolism pathway. We used volcano plots to find that the cardiac-specific changed metabolites were enriched to the sphingolipid metabolism pathway after TAVR. Besides, WGCNA algorithm was performed to reveal that arginine and proline metabolites could reflect left ventricle regression to some extent.

**Conclusion:**

This is the first study to reveal systemic and cardiac metabolites changed significantly in patients with AS after TAVR. Some altered metabolites involved in the arginine and proline metabolism pathway in the peripheral vein could predict left ventricle regression, which merited further study.

## 1. Introduction

Aortic stenosis (AS) is a progressive disease which is initially characterized by leaflet calcification and thickening. As the progression of AS, maladaptive cardiomyocyte apoptosis and diffused myocardial fibrosis result in heart failure and even multiorgan dysfunction [1]. Although cardiac magnetic resonance and echocardiography could reveal the early heart remolding, there were rare serum biomarkers that could reflect heart remolding [2, 3].

Metabolomics, as a well-constructed approach of systemic biology to offer a comprehensive description of low-molecular weight molecules participating in metabolism, is typically applied in investigating diseases mechanisms and discovering biomarkers [4]. Recent evidence revealed that cardiomyocyte metabolism would change in those patients with structural remolding and heart failure [5, 6]. Patients with symptomatic severe AS who often exerted remolding and heart failure apparently had changed the metabolic profile [7]. While, rare studies investigate the metabolite as a biomarker to reflect myocardial remolding in AS [3].

Transcatheter aortic valve replacement (TAVR) is a well-established strategy for treating patients with severely symptomatic AS who are at severe-, intermediate-, and even low-risk [8–11]. With the clinical indications of TAVR expanded, investigating the potential biomarker which reflected myocardial remolding could help to determine the optimal timing of TAVR in AS. Besides, the potential biomarker which could predict prognosis of TAVR in AS would help to make risk stratification. However, there were only few studies to utilize metabolomics to explore the potential biomarker in AS after TAVR [3, 12]. In addition, whether TAVR affects cardiac and systemic metabolite in patients with AS and the potential role of metabolite in predicting prognosis remained largely unknown.

Therefore, we performed this prospective study based on liquid chromatography-mass spectrometry (LC-MS) and gas chromatography-mass spectrometry (GC-MS) to (1) explore dynamic cardiac and systemic metabolic alteration in response to TAVR at an extremely early phase (within 30 minutes) and early phase (within 7 days) (2) and investigate their potential roles in predicting prognosis after TAVR, especially left ventricle (LV) function improvement.

## 2. Methods

### 2.1. Participants

A total of 59 consecutive patients diagnosed as symptomatic AS undergoing TAVR were enrolled in the study within 2017. All patients included in the present study signed the informed consent. This study was approved by the Institutional Review Board of West China Hospital, Sichuan University (Number of the approval 20140217).

### 2.2. TAVR Procedure

TAVR was performed through transfemoral access under general anesthesia by the same TAVR team as described in our previous study [13]. The endpoint of the results was defined and recorded according to Valve Academic Research Consortium-2 (VARC-2) criteria [14]. Transthoracic echocardiography was performed before discharge and at routine follow-up at 1 month, 3 months, 6 months, and 1 year after TAVR to evaluate the LV function.

### 2.3. Blood Sample Collection and Processing

**Figure 1:**
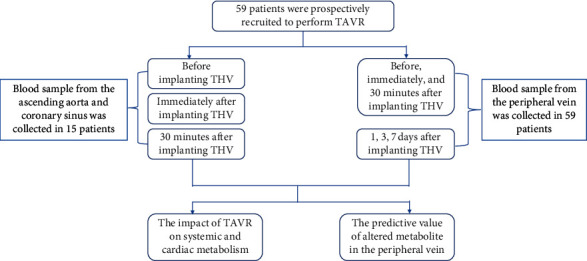
Brief protocol of the prospective study. TAVR, transcatheter aortic valve replacement; THV, transcatheter heart valve.

The protocol of the study is illustrated in Figure 1. In order to investigate the extremely early impact of TAVR on systemic and cardiac metabolism, blood sample from the ascending aorta, coronary sinus, and peripheral vein was collected at before, immediately, and 30 minutes after implanting the transcatheter heart valve (THV) in the hybrid operating room. Sample from the peripheral vein at 1, 3, and 7 days was also collected to explore the impact of TAVR on early systemic metabolism and to find the potential prognostic biomarker after TAVR.

Subsequently, these blood samples were centrifuged at 2,000 g for 10 minutes to pellet the cellular elements. The supernatant plasma was stored at −80°C until sample preparation for LC-MS and GC-MS, which were then sent for metabolomics detection in Dalian Institute of Chemical Physics of Chinese Academy of Sciences.

### 2.4. LC-MS Analysis

After thawing on the ice, a 50 *μ*L of blood sample was drawn and mixed with 200 *μ*L of methanol containing ISs (Supplemental Table s1). As previously reported, 180 *μ*L supernatant was drawn and underwent lyophilization treatment; then, the samples were redissolved in 80 *μ*L acetonitrile/water (1 : 4) solvent for LC-MS analysis [15]. Before analyzing the actual sample, a blank sample was used to balance the system. During the analysis of the actual sample, a quality control (QC) sample was inserted for every 10 samples to evaluate the data quality. The instrument and procedure of LC-MS analysis were described in the previous study [15].

### 2.5. GC-MS Analysis

Serum was thawed on the ice, and a 50 *μ*L of blood sample was drawn and mixed with 200 *μ*L of methanol containing ISs (Supplemental Table s1). As previously described, supernatants were lyophilization-treated for subsequent oximation and silylation reactions [16]. Then, the supernatant was obtained for GC-MS analysis. A QP 2010 Plus GC-MS system (Shimadzu, Japan) with a DB-5MS (Agilent Technologies, USA) was used. A QC sample was inserted for every 10 samples to evaluate the data quality. The instrument and procedure of GC-MS analysis were described in the previous study [16].

### 2.6. Metabolomics Analysis

The data of LC-MS were automatically peak-detected, sorted, and integrated by PeakView software (AB, SCIEX) to perform manual peak area integration on characteristic ions of known metabolites. The peak table was then exported to Excel Software for further analysis. With regard to GC-MS, a peak table was obtained using GC-MS postrun analysis (Shimadzu, Japan) based on a quantitative table. The mass spectrometry response of LC/GC-MS is corrected by using a virtual QC sample, which is described in a previous study [15, 17].

### 2.7. Statistics Analysis

The statistical analyses including PLS-DA analysis, fold change, and *t*-test analysis were applied to detect the significantly alerted metabolite profiling after THV implantation. The metabolites meeting the cutoff value of 1.2 for PLS-DA, 1.2 for fold change, and 0.05 for *p* value were regarded as significant.

The algorithm of STEM was performed to conclude the states of metabolites which varied along with time extension in this study [18]. In addition, WGCNA, as a robust tool for integrative network analysis, is widely used in the constructing complex network and exploring molecules in close relation to phenotypic alteration [19]. According to the WGCNA protocol, we detected hub molecules in response to prognostic parameter, such as left ventricular mass (LVM) and left ventricular mass index (LVMI), based on *R* software (https://www.r-project.org). Pathway analysis was established by MetaboAnalyst platform (http://www.metaboanalyst.ca) [20]. The data in this study were presented as the mean ± SD or median (IQR). A two-tailed *p* value < 0.05 was considered as significant, unless specifically indicated.

## 3. Results

### 3.1. Participants Information

Of the 59 patients with the average STS score of 8.04% enrolled in the present analysis, 26 were male (Table 1). The maximum transaortic velocity and mean transaortic gradient decreased significantly after TAVR from 5.0 ± 0.8 m/s to 2.3 ± 0.6 m/s and 62.9 ± 20.6 mmHg to 13.3 ± 7.7 mmHg, respectively. Consistently, the LVMI decreased markedly from 177.0 ± 56.7 g/m^2^ to 129.3 ± 36.9 g/m^2^, and the left ventricle ejection fraction (LVEF) increased markedly from 50.7 ± 15.2 to 64.3 ± 7.3%. The level of BNP also decreased dramatically from 3665 (1379–10395) pg/ml to 455.5 (157–1014) pg/ml after heart function improvement (Supplemental Figure s1, all *p* value<0.05).

### 3.2. Extremely Early Alteration of Metabolites after TAVR

#### 3.2.1. Metabolites in the Ascending Aorta

**Figure 2:**
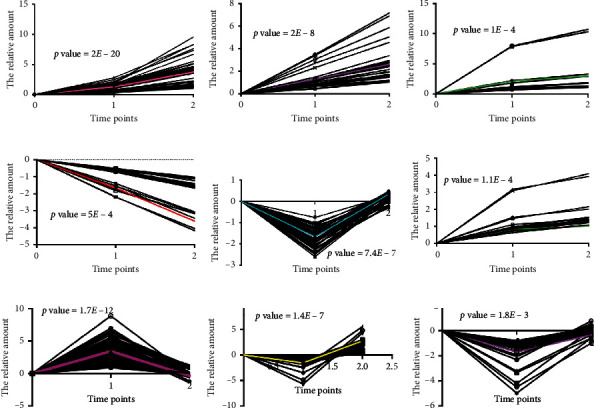
The extremely early altered metabolic pattern from the ascending aorta, coronary sinus, and peripheral vein after TAVR. (a–d) The altered metabolic pattern from the ascending aorta; (e, f) the altered metabolic pattern from the coronary sinus; (g–i) the altered metabolic pattern from the peripheral vein.

**Figure 3:**
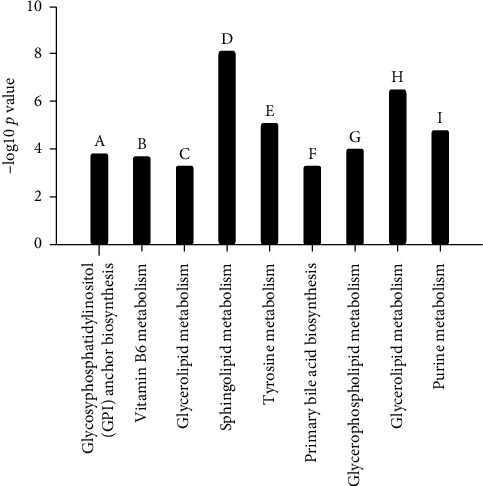
The extremely early altered metabolism pathway responding to TAVR. (a–d) The involved metabolism pathway based on altered metabolite from the ascending aorta; (e, f) the involved metabolism based on altered metabolite from the coronary sinus; (g–i) the involved metabolism based on an altered metabolite from the peripheral vein.

According to the PLS-DA analysis, an obvious separation trend was detected after THV implantation in the ascending aorta (Supplemental Figure s2A, *p* < 0.05). Based on the STEM algorithm after filtering the significantly varied metabolites, these changed metabolites presented four disparate patterns, including three elevated ones (Figures 2(a)–2(c)) and a descending one (Figure 2(d)). These metabolites in Figure 2(a) could be enriched to the glycosylphosphatidylinositol (GPI) anchor biosynthesis pathway (Figure 3, column A, *p* < 0.05), while the molecules in Figures 2(b)–2(d) were mapped to the vitamin B6 metabolism pathway (Figure 3, column B, *p* < 0.05), glycerolipid metabolism pathway (Figure 3, column C, *p* < 0.05), and sphingolipid metabolism pathway (Figure 3, column D, *p* < 0.05), respectively.

#### 3.2.2. Metabolites in the Coronary Sinus

We also perform PLS-DA analysis to investigate the metabolite alteration in the coronary sinus, and we found that there was a significant separation trend after THV implantation (Supplemental Figure s2B, *p* < 0.05). The extremely early changed metabolite of the coronary sinus showed two different patterns, one was descending immediately after TAVR and then ascending (Figure 2(e)), while the other was continually ascending (Figure 2(f)), which involved in the tyrosine metabolism and primary bile acid biosynthesis pathway, respectively (Figure 3, columns E and F, all *p* < 0.05).

#### 3.2.3. Metabolites in the Peripheral Vein

PLS-DA analysis was also carried out to explore the metabolite changes in the peripheral vein, and a significant separation trend was detected (Supplemental Figure s2C, *p* < 0.05). It displayed three patterns, one was ascending first and then descending (Figure 2(g)), while the others presented descending first and then ascending (Figures 2(h) and 2(i)), which were involved in the glycerophospholipid metabolism (Figure 3, column G, *p* < 0.05), glycerolipid metabolism (Figure 3, column H, *p* < 0.05), and purine metabolism pathway (Figure 3, column I, *p* < 0.05), respectively.

#### 3.2.4. Cardiac-Derived Metabolites after TAVR

**Figure 4:**
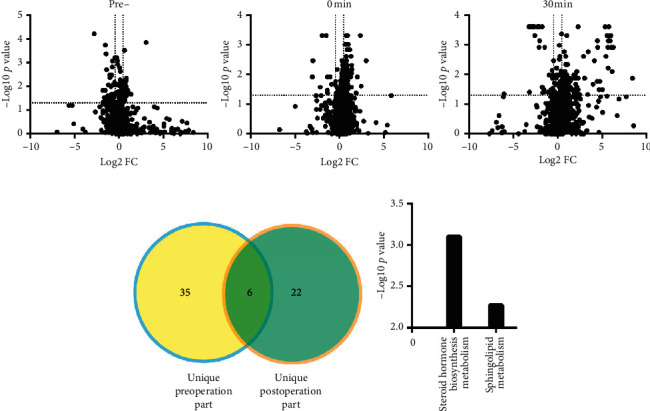
The specific cardiac metabolism before and after TAVR. (a) Volcano analysis regarding the difference between the ascending aorta and coronary sinus at before, immediately, and 30 minutes after implanting the transcatheter heart valve (THV); (b) the number of unique cardiac metabolite before and after THV; (c) the involved metabolism pathway before and after THV.

Although significant alteration of metabolites was found in blood samples from the ascending aorta, coronary sinus, and peripheral vein after TAVR, while their metabolic alteration could not reflect cardiac specific metabolites alteration directly. The blood suppling the heart originates from the base of the ascending aorta, and after substance exchange, they eventually return to the right atrium through the coronary sinus. Thus, as illustrated in Figure 4(a), volcano plots were constructed to explore the cardiac specific metabolites by calculating differentially expressed molecules between the ascending aorta and coronary sinus before and after TAVR. Among these cardiac specific metabolites, 35 unique metabolites before TAVR could be mapped to the steroid hormone biosynthesis pathway, while 22 unique molecules post-TAVR were enriched to the sphingolipid metabolism pathway (Figures 4(b) and 4(c)).

### 3.3. Early Metabolic Alteration in the Peripheral Vein after TAVR

Except for the extremely response of metabolite to TAVR in patients with AS, we also collected 1 day, 3 days, and 7 days blood samples in the peripheral vein to investigate the early metabolic alteration after TAVR. Six disparate patterns were identified from changed metabolites in the early phase of the peripheral vein since 1 day after TAVR. Metabolites in pattern A (Supplemental Figure s3A) decreased 1 day after TAVR and then remained stable at 3 and 7 days, which were involved in the purine metabolism pathway (Supplemental Figure s4, column A, *p* < 0.05). These molecules in pattern B (Supplemental Figure s3B) increased up to 3 days and then decreased at 7 days after TAVR, which participated in primary bile acid biosynthesis (Supplemental Figure s4, column B, *p* < 0.05). While the metabolites in Supplemental Figure s3C increased gradually up to 7 days, which were engaged in the glycerolipid metabolism pathway (Supplemental Figure s4, column C, *p* < 0.05). Metabolites in Supplemental Figure s3D were increased significantly starting from 1 day up to 3 days and remaining stable after TAVR, which could be enriched to amino sugar and nucleotide sugar metabolism (Supplemental Figure s4, column D, *p* < 0.05). In addition, elevated molecules after 1 day, which experienced sudden decrease subsequently (Supplemental Figures s3E and F), were engaged in one carbon pool by folate and alanine and the aspartate and glutamate metabolism pathway (Supplemental Figure s4, column E and F, *p* < 0.05).

### 3.4. Potential Biomarkers in the Peripheral Vein to Predict LV Regression

In addition to identifying the early metabolites alteration after TAVR in the peripheral vein, we do further research to find the potential biomarkers in the peripheral vein that could predict prognosis in patients undergoing TAVR. Considering the few events of 30-day or 1-year mortality, we concentrate the potential biomarkers on LV regression which may serve as risk stratification or timing of TAVR in the future. WGCNA algorithm was performed to reveal hub molecules highly correlated with 1 year LV regression based on blood samples acquired on 1^st^ day, 3^rd^ day, and 7^th^ day after TAVR. Accordingly, these crucial modules including MEorgane module for 1^st^ day, MEbrown module for 3^rd^ day, and MElightyellow for 7^th^ day were obtained and are illustrated in Supplemental Figure s5A, with the essential metabolites inside exhibiting a obviously negative relationship to LV regression. Then, shared metabolites of these three modules were identified by Venn figure (Supplemental Figure s5B) and significantly mapped to arginine and proline metabolites by enrichment analysis (Supplemental Figure s5C, *p* value<0.05). Besides, the representative metabolite involved in the arginine and proline metabolism pathway increased remarkably on 1^st^ day, 3^rd^ day, and 7^th^ day after TAVR, comparing to preprocedure (Supplemental Figure s5D, *p* value<0.05).

## 4. Discussion

AS caused a relative myocardial ischemia due to myocardial oxygen supply and demand mismatch from reduced coronary flow reserve because of long-term pressure overload. The hypertrophic cardiomyocyte, increased wall stress, systolic dysfunction, and diastolic dysfunction were also seen in patients with AS [1]. These structural and functional alteration of the heart also resulted in derangement of myocardial metabolism including decreased mitochondrial energy production, insulin resistance, and perturbations in amino acid, lipid, and nucleotide metabolism [7]. Heart failure was a common manifestation in patients with severe AS. Previous study demonstrated that patients with heart failure showed cardiac metabolism alteration presenting suppression of oxidative phosphorylation with reduced utilization of fatty acid and in combination with increased glucose consumption [21]. However, previous studies focused on the metabolite alteration including fatty acid, glucose, amino acid, and ketone bodies in failing status due to pressure overload, how the metabolite changes, and which metabolic pathway responds when relieving pressure overload remains unclear.

Patients with AS who underwent TAVR were a natural model to find the changed metabolites after pressure overload alleviation. Thus, in the present study, we used LC/GC-MS-based metabolomics profiling techniques to explore the metabolites changes after TAVR. To be the best knowledge of us, this was the first study to reveal that TAVR could result in the alteration of systemic and cardiac metabolism in AS patients. We found that the metabolic pattern of the ascending aorta and peripheral vein changed significantly immediately and 30 minutes after THV implantation compared with preprocedure, which we defined as an extremely early change. The metabolite in the ascending aorta mapped to glycosylphosphatidylinositol (GPI) anchor biosynthesis, vitamin B6 metabolism, glycerolipid metabolism, and sphingolipid metabolism, while the metabolite in the peripheral vein enriched to glycerophospholipid metabolism, glycerolipid metabolism, and purine metabolism. However, although metabolite in the ascending aorta and peripheral vein after TAVR is mapped to different pathways, they both mainly enriched to lipid metabolism in our study, which revealed an increased demand for lipid metabolism after alleviation of pressure overload. Previous study demonstrated a shift in fuel metabolism from fatty acid oxidation to glucose in the failing heart [21]. Therefore, when heart failure is recovered, the fuel metabolism in the heart would redepend on fatty acid oxidation. Besides, a hypercatabolic state has been reported in patients with heart failure [22]. We found that GPI anchor biosynthesis, vitamin B6 metabolism, and purine metabolism increased after TAVR, which revealed a hypercatabolic state toward the anabolic state after heart function improvement.

In consistence with an extremely early change in the peripheral vein, the early change of the metabolic pattern in the peripheral vein which means 1 day, 3 days, and 7 days after TAVR also altered significantly. The increase of primary bile acid biosynthesis and glycerolipid metabolism showed an active lipid metabolism after TAVR, which was similar to the findings in the extremely early change of metabolites in the peripheral vein after TAVR. Additionally, the increase of amino sugar and nucleotide sugar metabolism and one carbon pool by folate also represented an anabolic state rather than the catabolic state after TAVR, which was also consistent with that in the extremely early change in the peripheral vein. These changes of the metabolism pathway revealed a shift of fuel redepend on fatty acid oxidation and a predominant anabolic state when remolding of the heart improved.

Except for the impact of TAVR on systemic metabolism, we also investigated the cardiac specific metabolism before and after TAVR to know how TAVR affects cardiac metabolism. We utilized blood samples from the coronary sinus and ascending aorta, which reflected the beginning and ending of the cardiac circulatory to identify specific cardiac metabolite. And we found that the steroid hormone biosynthesis pathway was predominant in patients before TAVR. Previous studies reported that steroid hormone including mineralocorticoid hormone exerted adverse effects on the homeostasis of the cardiovascular system resulting in occurrence of heart failure [23]. The present study also confirmed that the steroid hormone pathway was involved in the cardiac remodeling when the pressure overload increases due to AS. However, the sphingolipid metabolism pathway was predominant in patients after TAVR. Sphingosine-1-phosphate increased after TAVR, which enriched to the sphingolipid metabolism pathway in the present study. The relationship between sphingosine-1-phosphate and heart failure was described elsewhere. Polzin et al. found that plasma sphingosine-1-phosphate concentration correlated with impaired LV function, and the cardiac sphingosine-1-phosphate receptor was downregulated in the isoproterenol-induced heart failure model [24]. In addition, Deshpande et al. revealed that sphingosine-1-phosphate could protect against heart failure through activating STAT3 [25]. Thus, the sphingosine-1-phosphate concentration increased after alleviation of pressure overload may also enhance the evidence that sphingosine-1-phosphate may be a potential therapy in patients suffering from heart failure.

As the indication of TAVR extended, increasing studies were performed to find the predictor of long-term prognosis. However, limited biomarkers were proposed as a predicting long-term outcome [2, 3, 26]. In the present study, we found altered metabolites responding to TAVR in the peripheral vein in 1 day, 3 days, and 7 days after TAVR could reflect LVM regression. The enrichment analysis based on the cochanged metabolites of three follow-up times revealed tyrosine metabolism and arginine and proline metabolism involved in the LVM regression. As previous articles described, arginine and proline metabolism contribute to the synthesis of nitric oxide (NO), which was essential for regulating vascular tone and hemodynamics [27]. Patients with heart failure were associated with a reduced plasma arginine level as reported elsewhere, which suggested that decrease of arginine and proline metabolism participated in the progression of heart failure [28]. There was increasing evidence indicating that oral intake of arginine would decrease vascular resistance, improve endothelium-dependent vasodilation, and increase cardiac output [29, 30]. Besides, supplementation of arginine also protected myocardium against reoxygenation injury [31]. Thus, the increased arginine and proline metabolism responded to alleviation of AS, and subsequently decreasing myocardial stress might be accounted for the improvement of LVM regression. Additionally, data mining and analysis of GSE51472 database also figured out that endothelial nitric oxide synthase (eNOS) which generates NO was lower in patients with AS (Supplemental Figure s5E), which in turn prove that the findings of TAVR could increase arginine and proline metabolism in patients suffering from AS.

## 5. Limitations

Several limitations should be considered when interpreting the results of this research. The sample size was limited, and only discovery cohort was performed, albeit collecting blood samples from the ascending aorta, coronary sinus, and peripheral vein. This limitation made these findings of the present study to be considered more suitable as generating hypothesis. Second, the difference of post-TAVR-related medical behavior including implanting PPM and transfusion due to conduction disturbance and bleeding complication, respectively, might influence the level of metabolite. Even a single center study could not eliminate such bias.

## 6. Conclusion

The systemic and cardiac metabolic pattern altered significantly in patients suffering from AS after THV implantation, and sphingolipid metabolism was unique in cardiac metabolism after TAVR. While increased arginine and proline metabolism due to alleviation of pressure overload were related with the improvement of LVM regression. More studies are urgently needed to investigate the role of metabolomics in patients with AS undergoing TAVR.

## Figures and Tables

**Table 1:** Clinical characteristics of participants.

Characteristics	*N* = 59 (peripheral vein)	*N* = 15 (three source)

Male (*n*, %)	26 (44.1%)	8 (53.3%)
Age (yrs)	73.3 ± 5.5	74.1 ± 4.9
STS (%)	8.04 ± 4.6	9.0 ± 3.4
NYHA III/IV	55 (93.2%)	14 (93.3%)
Body mass index (kg/m^2^)	22.4 ± 3.3	22.9 ± 4.1
Hypertension (*n*, %)	24 (40.7%)	12 (80.0%)
Diabetes (*n*, %)	5 (8.5%)	1(6.7%)
Chronic obstructive pulmonary disease (*n*, %)	25 (42.4%)	10 (66.7%)
Coronary artery disease (*n*, %)	26 (44.1%)	5 (33.3%)
Peripheral artery disease (*n*, %)	40 (67.8%)	8 (53.3%)
Cerebrovascular disease (*n*, %)	11 (18.6%)	3 (20%)
Chronic kidney disease (*n*, %)	11 (18.6%)	3 (20%)
Maximum transaortic velocity (m/s)	5.0 ± 0.8	4.9 ± 0.7
Mean transaortic gradient (mmHg)	62.9 ± 20.6	64.1 ± 17.1
Left ventricular ejection fraction (%)	50.7 ± 15.2	48.1 ± 13.2
Left ventricular mass (g)	296.0 ± 97.6	310.8 ± 78.8
Left ventricular mass index (g/m^2^)	177.0 ± 56.7	183.6 ± 43.6
Preprocedure NT-ProBNP (pg/ml)	3665.0 (1056–12209.3)	2572.0 (1999.0–8357.0)
30-day mortality	1 (1.7%)	1 (6.7%)
1-year mortality	3 (5.1%)	3 (20%)

NYHA, New York Heart Association.

## Data Availability

The data used to support the findings of this study are available from the corresponding author upon request.

## Supplementary Materials

Supplementary Materials Supplemental Figure s1: the impact of TAVR on heart function and hemodynamic parameter in patients with aortic stenosis. EF, left ventricular ejection fraction; LVM, left ventricular mass; LVMI, left ventricular mass index; pean, mean transaortic gradient; Vmax, maximum transaortic velocity. Supplemental Table s1: internal standard in liquid chromatography-mass spectrometry and gas chromatography-mass spectrometry.
