# Supplementary material for: Metabolic Modulation and Potential Biomarkers of the Prognosis Identification for Severe Aortic Stenosis after TAVR by a Metabolomics Study
Source: Cardiol Res Pract. 2020 Oct 28;2020:3946913. doi: 10.1155/2020/3946913 (PMC7649585; doi:10.1155/2020/3946913)
Supplement: Supplementary Materials — Supplemental Figure s1: the impact of TAVR on heart function and hemodynamic parameter in patients with aortic stenosis. EF, left ventricular ejection fraction; LVM, left ventricular mass; LVMI, left ventricular mass index; pean, mean transaortic gradient; Vmax, maximum transaortic velocity. Supplemental Table s1: internal standard in liquid chromatography-mass spectrometry and gas chromatography-mass spectrometry. [file 3946913.f1.zip › 3946913.f1/Supplemental Figures.pdf]

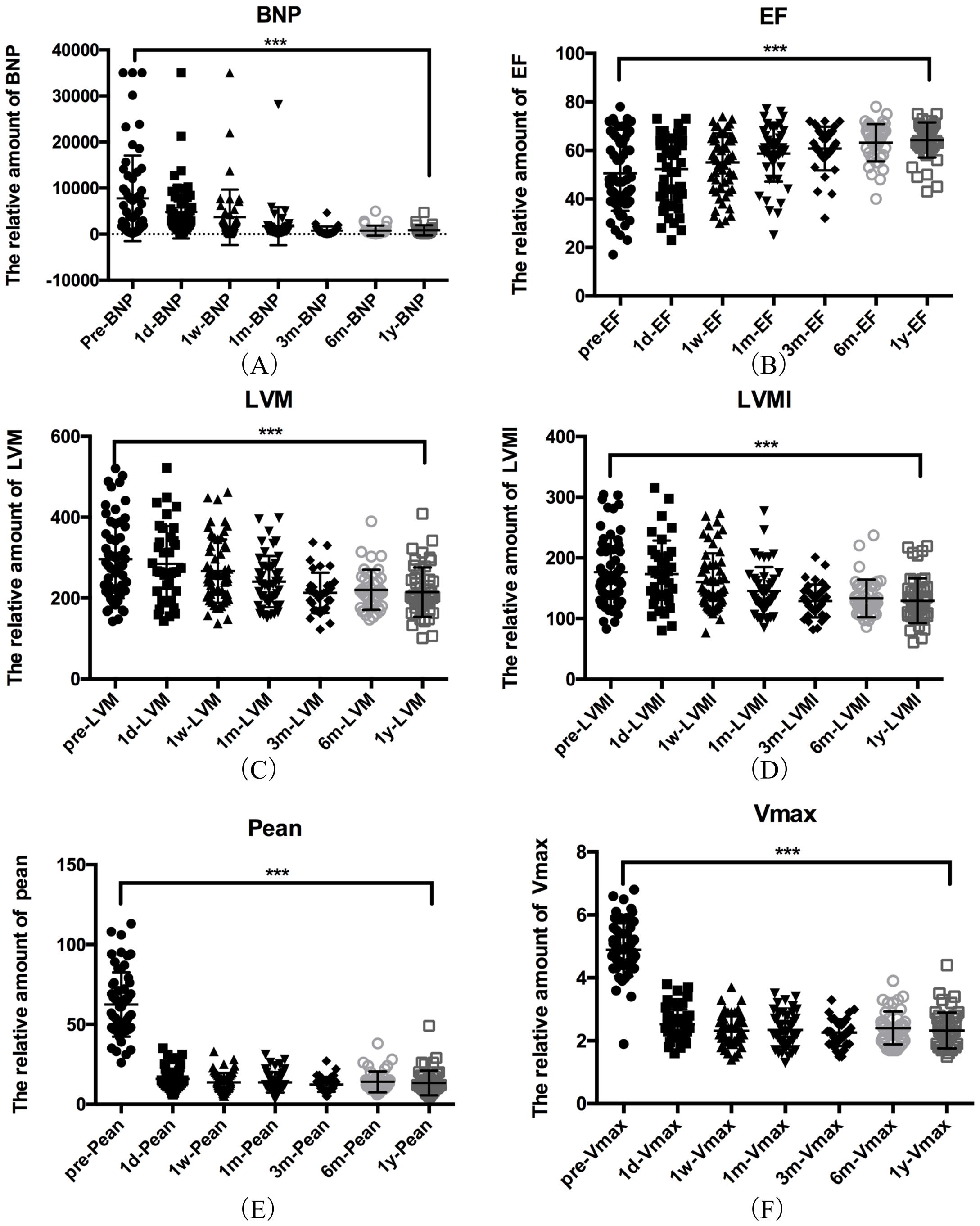

Supplemental Figure s1 The impact of TAVR on heart function and hemodynamic parameter in patients with aortic stenosis
EF= left ventricular ejection fraction; LVM= left ventricular mass; LVMI= left ventricular mass index; Pean= mean transaortic gradient; Vmax= maximum transaortic velocity

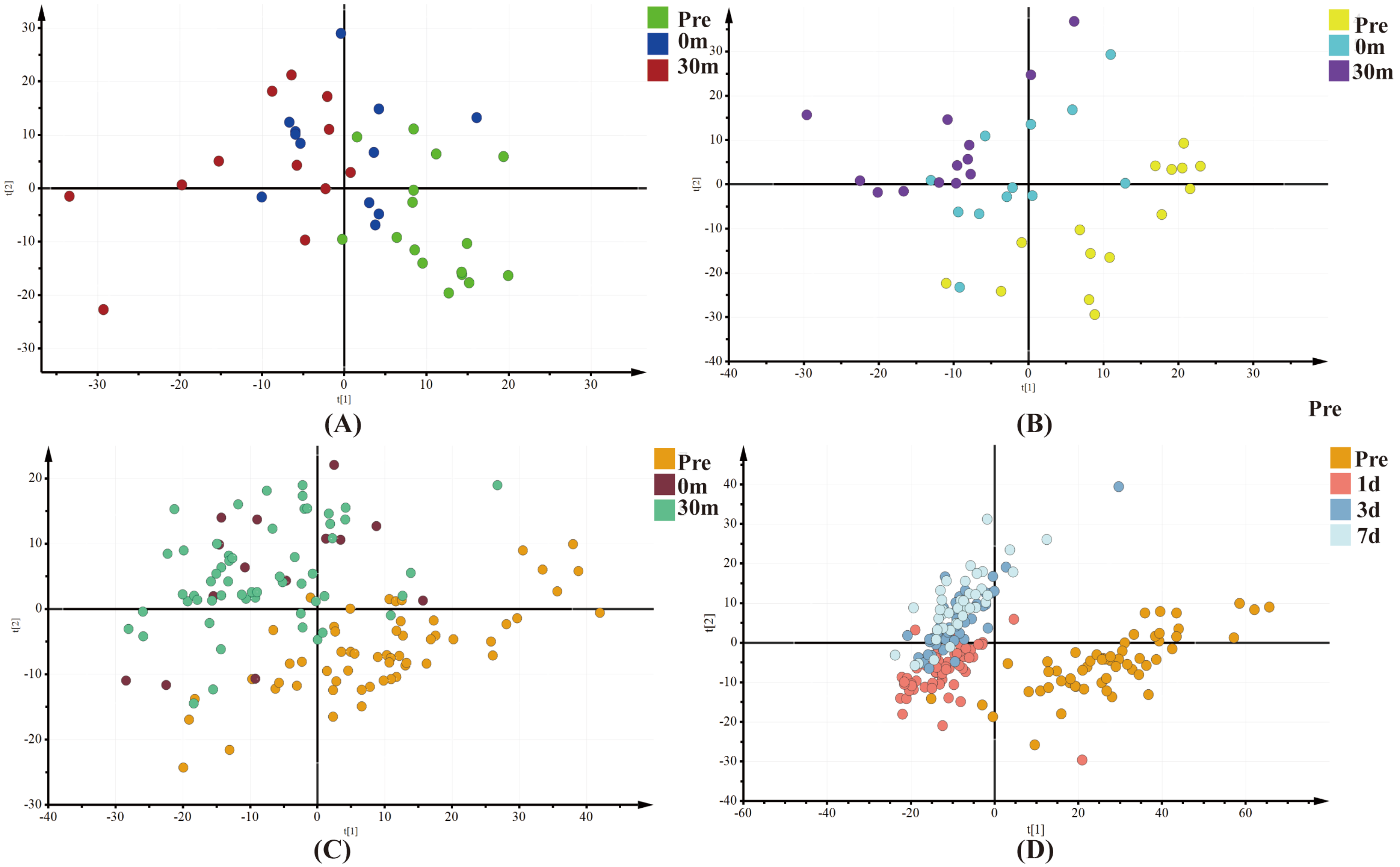

Supplemental Figure s2 TAVR affected metabolite from ascending aorta, coronary sinus and peripheral vein in patients with aortic stenosis

pre means the blood sample before procedure; 0m means the blood sample immediately after implanting transcatheter heart valve (THV); 30m means the blood sample 30 minutes after implanting THV; 1d means the blood sample 1 day after implanting THV; 3d means the blood sample 3 days after THV; 7d means the blood sample 7days after THV.

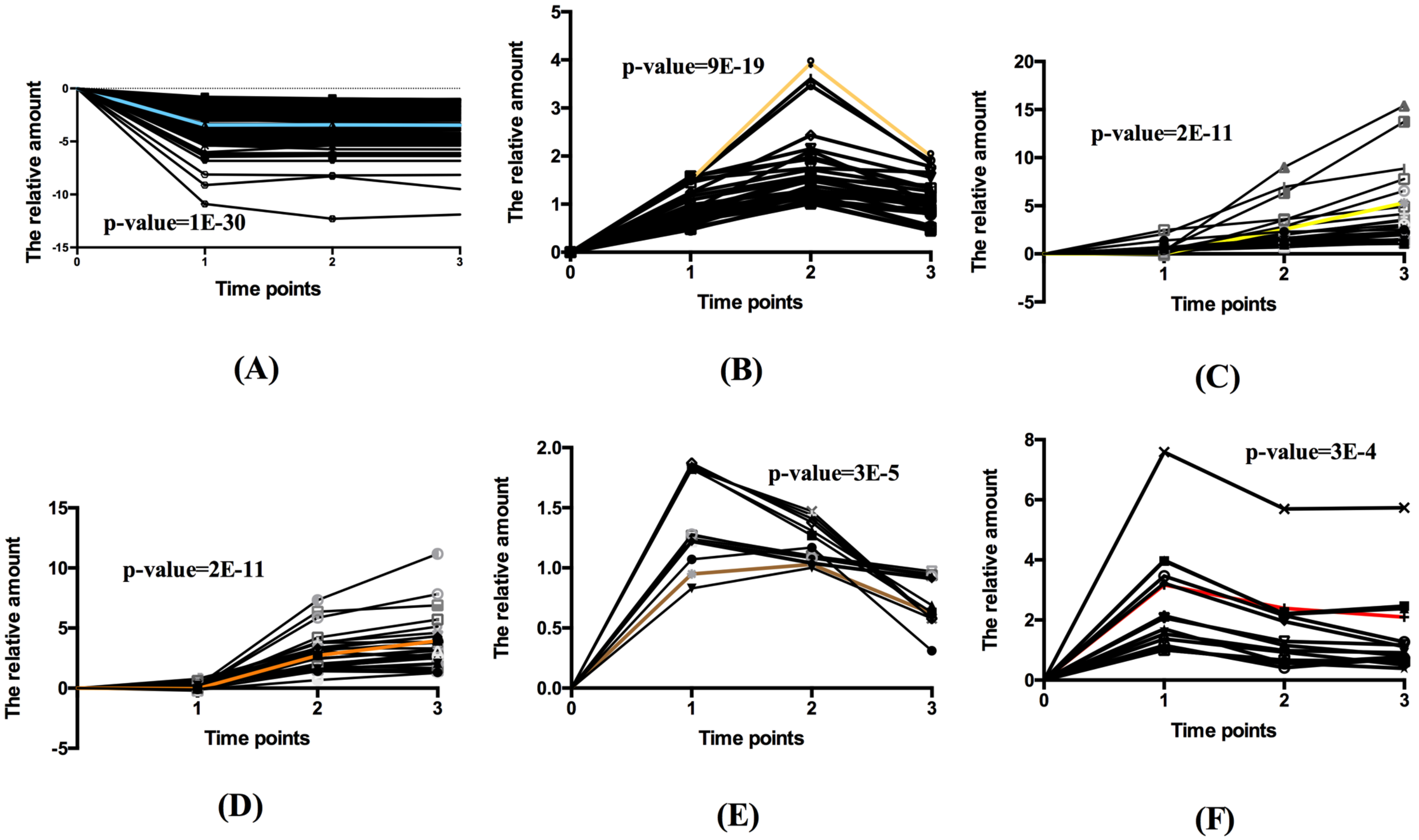

Supplemental Figure s3 The early altered metabolic pattern from peripheral vein after TAVR
Panel A-F indicates the early altered metabolic pattern from peripheral vein after TAVR

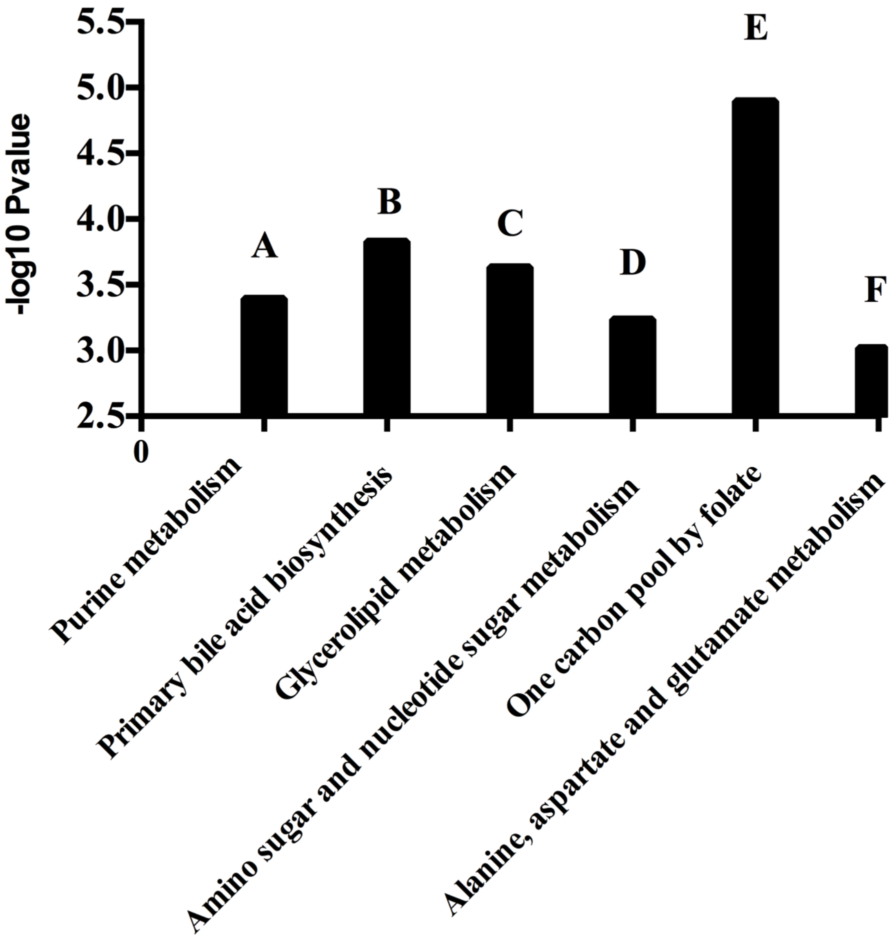

Supplemental Figure s4 The early altered metabolism pathway responding to TAVR
Panel A-F indicates the early altered metabolism pathway from peripheral vein after TAVR

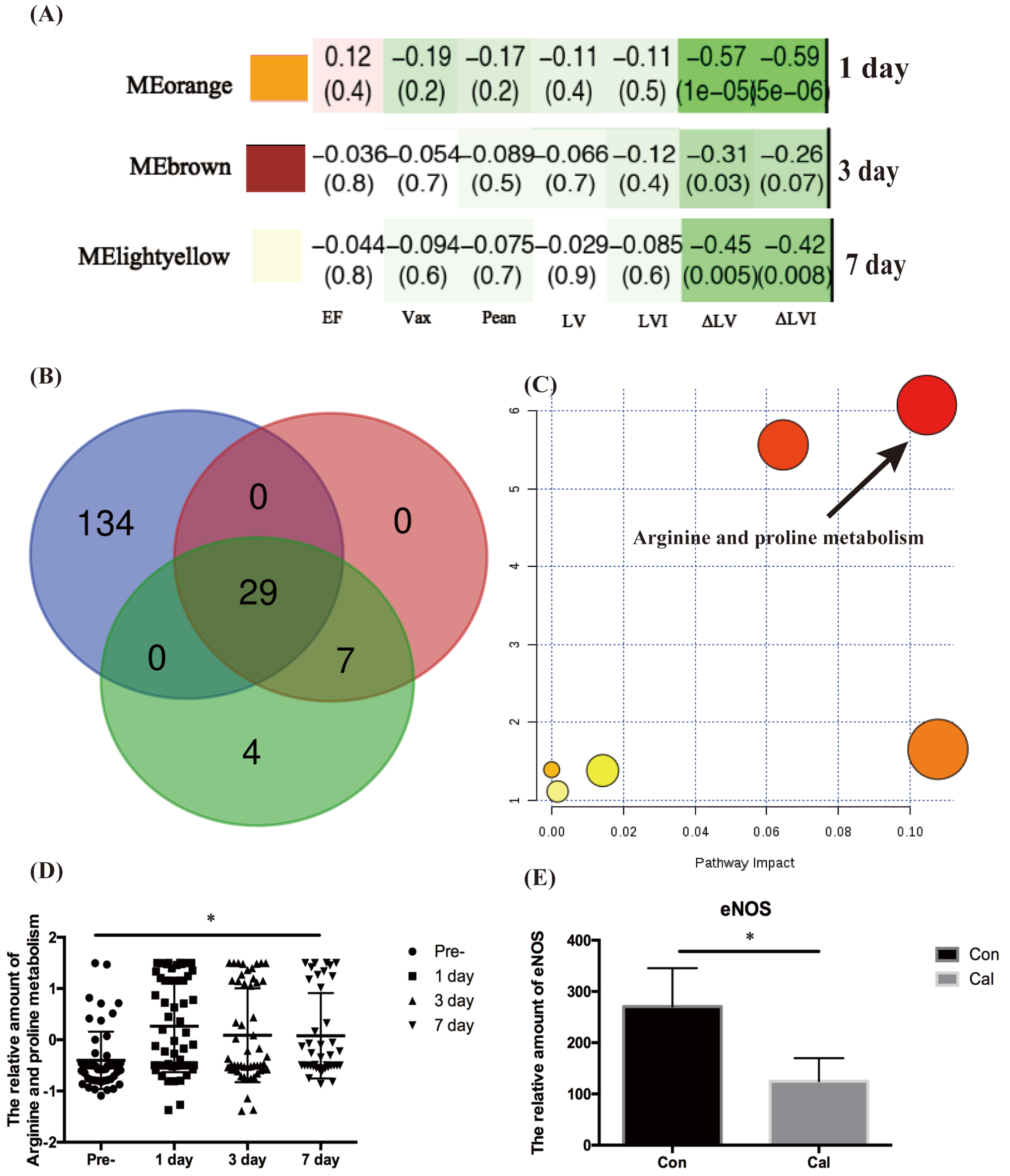

Supplemental Figure s5 The predictive value of early changed metabolite from peripheral vein on left ventricle remolding in patients with aortic stenosis after TAVR

Panel A indicates the potential metabolic profile at three time points predicting prognosis; panel B indicates the predominant changed metabolite at three time points; panel C indicates the metabolism pathway in which the predominant altered metabolite involved; panel D indicates the altered metabolism pathway after TAVR; panel E indicates the level of eNOS was lower in patients with aortic stenosis based on the analysis of GSE1472 database.
