# Supplementary material for: Metabolic Modulation and Potential Biomarkers of the Prognosis Identification for Severe Aortic Stenosis after TAVR by a Metabolomics Study
Source: Cardiol Res Pract. 2020 Oct 28;2020:3946913. doi: 10.1155/2020/3946913 (PMC7649585; doi:10.1155/2020/3946913)
Supplement: Supplementary Materials — Supplemental Figure s1: the impact of TAVR on heart function and hemodynamic parameter in patients with aortic stenosis. EF, left ventricular ejection fraction; LVM, left ventricular mass; LVMI, left ventricular mass index; pean, mean transaortic gradient; Vmax, maximum transaortic velocity. Supplemental Table s1: internal standard in liquid chromatography-mass spectrometry and gas chromatography-mass spectrometry. [file 3946913.f1.zip › 3946913.f1/Supplemental table s1.doc]

Supplemental table s1 Internal standard in liquid chromatography-mass spectrometry and gas chromatography-mass spectrometry

Internal standard

Liquid Chromatography-Mass Spectrometry

Lyso-phosphatidylcholine (LPC) 19:0

Camitine C2:0-d3

Camitine C10:0-d3

Camitine C16:0-d3

Free fatty acid (FFA) 16:0-d3

FFA 18:0-d3

Cholic acid-d4

Chenodeoxycholic acid-d4

Leucine-d3

Phenylalanine-d5

Tryptophan-d5

Gas Chromatography-Mass Spectrometry

Tridecanoic acid

Succinic acid-d4

Citric acid-d4

Valine-d8

Phenylalanine-d5
